# Knowledge on Palliative Care and Associated Factors among Nurses in Ethiopia: A Systematic Review and Meta-Analysis

**DOI:** 10.1155/2021/5557947

**Published:** 2021-04-24

**Authors:** Addisu Getie, Adam Wondmieneh, Melaku Bimerew, Getnet Gedefaw, Asmamaw Demis

**Affiliations:** ^1^Department of Nursing, College of Health Sciences, Woldia University, P.O.Box: 400, Woldia, Ethiopia; ^2^Department of Midwifery, College of Health Sciences, Woldia University, P.O.Box: 400, Woldia, Ethiopia

## Abstract

**Background:**

Palliative care is a multidisciplinary team-based care for patients facing life-threatening illness and their families which addresses their physical, psychological, social, and spiritual needs to improve the quality of care. There is a strategy for an increase in palliative care services by integrating with the healthcare system. Therefore, this systematic review and meta-analysis was aimed to assess the overall pooled prevalence of nurses' knowledge towards palliative care in Ethiopia.

**Method:**

PubMed/MEDLINE, HINARI, EMBASE, Scopus, Google Scholar, and African Journals OnLine (AJOL) were the databases used to search for articles. Cochrane *I*^2^ statistics and Egger's test were done to check heterogeneity and publication bias, respectively. Subgroup analysis by region, study period, and sample size was done due to the presence of heterogeneity. Sensitivity analysis was also done to detect the presence or absence of an influential study.

**Result:**

Nine studies with a total of 2709 study participants were included in the final analysis. The overall pooled prevalence of nurses' knowledge towards palliative care was 45.57% (95% CI: 35.27–55.87). Educational status and palliative care training were significantly associated factors with the level of nurses' knowledge towards palliative care. B.S. degree holder nurses (AOR = 3.01; 95% CI: 1.50–6.02) and nurses who had palliative care training (AOR = 4.64; 95% CI: 2.37–9.08) were found to be significantly associated factors with the nurses' level of knowledge.

**Conclusion:**

More than half of nurses had poor knowledge of palliative care. Educational status of nurses and palliative care training were significantly associated factors with the nurses' level of knowledge about palliative care. Therefore, palliative care training and improving nurses' careers through continuous professional development should be focused on regularly to improve nurses' knowledge about palliative care.

## 1. Introduction

Palliative care is the science of promoting the quality of life patients (adults and children) and their families, which is effective in the patient's late life and useful in relieving suffering from life-threatening terminal illness through early identification, correct assessment, treatment of pain, and other problems. It uses a team approach to address the needs of the patients and their families, including counseling, positively influencing the course of illness, and managing distressing clinical complications [1–3]. Palliative care has its standards that describe the systems and enablers necessary to deliver high-quality clinical care, and initial and ongoing assessments incorporate a patient's physical, psychological, cultural, social, and spiritual experiences and needs. The standards of palliative care also describe the explanation regarding quality management, quality improvement, and benchmarking [4]. Globally, 56,840,123 people require palliative care. It was needed for approximately 45% of all deaths in 2017 and 40% of patients in need of palliative care were aged 70 years and above [1]. Palliative care can benefit approximately 75% of people approaching the end of life. The growing number of older people and an increase of chronic illnesses in many countries including low-income settings make palliative care the center of focus [5]. Staff nurses should appropriately qualify and engage in continuous professional development and are supported in their role to provide palliative care. There are different roles of nurses regarding palliative care. These include being available on the patient's side. Nurses generally spent their time mostly with their patients to give basic care, even though the patient received care from other health professionals [6]. Nurses need to be equipped with palliative care knowledge to provide optimal care for patients suffering from chronic and serious illness and their families to improve their quality of life. However, a lack of nurses' knowledge regarding palliative care, which might be a lack of education in pain and symptom management and communication about goals of care, can result in a suboptimal and high cost of care [7]. Different studies revealed that the nurses' level of knowledge about palliative care was 44.5% in New York [8], 44.5% in Greece [9], 40% in Mongolia [10], and 43.5% in Pakistan [11], which was below the standards of the board [12]. As previous studies showed, lack of palliative care education, not participating in important aspects of health care, lack of experience, lack of proper training, few specialization units, and poor arrangement of a career ladder for nurses were found to be contributing factors for the poor knowledge of nurses about palliative care [8, 10, 12, 13]. Access to palliative care is a key quality metric that all health care organizations should improve [14].

Nurses have a prominent role in providing palliative care. Nurses must provide quality care to terminally ill and chronically sick patients. To improve the quality of life of the patients, nurses must have good knowledge of palliative care. However, little is known regarding palliative care preparedness among Ethiopian nurses. Thus, this systematic review and meta-analysis was designed to identify the level of knowledge about palliative care and associated factors among nurses in Ethiopia.

## 2. Methods and Materials

### 2.1. Study Protocol

Preferred Reporting Items for Systematic Review and Meta-analysis (PRISMA) checklist for reporting of findings was used in this systematic review and meta-analysis, as shown in the supplementary file (Table S1) [15].

### 2.2. Databases and Search Strategies

In this systematic review and meta-analysis, PubMed/MEDLINE, HINARI, EMBASE, Google scholar, and African Journals OnLine (AJOL) searching databases were used. The published and unpublished articles from the repositories of Ethiopian universities were also searched. The searching date ranged from September 1 to September 23, 2020. Studies reporting knowledge on palliative care and associated factors among nurses in Ethiopia were included in the analysis. “Knowledge,” “awareness,” “palliative care,” “end of life care,” “caring terminally ill,” “factors,” “associated factors,” “determinant factors,” “nurses,” “hospital-based nurses,” and “Ethiopia” were searching items using “AND” and “OR” Boolean operators strings (Table 1).

### 2.3. Screening and Eligibility of Studies

After searching for accessible articles, all retrieved articles were exported into the “EndNote reference software version 8 (Thomson Reuters, Stamford, CT, USA) citation manager”. Then all articles were sorted and the duplications were removed. Two investigators (AG and AD) independently evaluated each study by title and abstracts using predetermined inclusion criteria. The eligibility of all studies for the final analysis was also assessed by critically reviewing the full text of the selected studies. In the extraction sheet, the first name of the authors, publication year, region where the study was conducted, sample size, study design, study period, level of knowledge, and factors affecting the level of knowledge were extracted. There was no discrepancy between the authors during the process of extraction, evaluation, and reviewing of the articles.

### 2.4. Inclusion and Exclusion Criteria

All published and unpublished cross-sectional studies conducted from January 1, 2010, to September 23, 2020, on nurses' level of knowledge about palliative care and associated factors in Ethiopia were included in this systematic review and meta-analysis. Articles that did not report outcome variables, different trials, review articles, case reports, and news were excluded from this review. Furthermore, qualitative studies, interventional studies, and studies without full text were also excluded from the final analysis.

### 2.5. Outcome Measurement of the Study

This systematic review and meta-analysis has two outcomes. The first outcome was the level of nurses' knowledge on palliative care, which was measured by the mean score of knowledge (good knowledge/poor knowledge) using knowledge assessing items. Thus, nurses who scored mean and above mean of knowledge assessing items were considered knowledgeable, whereas nurses who scored below the mean score of knowledge assessing items were considered not knowledgeable. The second outcome was the factors affecting the knowledge of nurses regarding palliative care.

### 2.6. Quality Assessment

The quality of the studies included in this systematic review was assessed using the Newcastle-Ottawa Scale (NOS) for cross-sectional studies [16] by two independent authors (AG and AD). The studies were included based on the Newcastle-Ottawa Scale's quality assessment criteria. The methodological quality, comparability, outcomes, and statistical analysis of the studies were the assessment tools used to declare the quality of studies. Studies scored on a scale of >7 out of 10 were considered as achieving high quality. All authors independently assessed the articles for consideration and inclusion in the final analysis.

### 2.7. Data Processing and Analysis

The inverse-variance random-effects model at 95% Cl was used to weigh the pooled prevalence of the level of knowledge regarding palliative care and its associated factors among nurses in Ethiopia [17]. We used a Microsoft Excel spreadsheet to extract and clean the data. Then, the extracted data were exported to STATA version 11 statistical software for analysis. Cochrane Q-test and *I*^2^ with its corresponding *p* values were used to assess the heterogeneity of studies [18]. Subgroup analysis by a region where the study was conducted, the study period, and the sample size was carried out to examine the source of heterogeneity. Sensitivity analysis was carried out to check the presence of an influential study. Egger's test was done to detect the presence of publication bias and was presented with a funnel plot [19]. A log odds ratio was used to decide the association between associated factors and the nurses' level of knowledge about palliative care. A statistical test with a *p* value < 0.05 was considered statistically significant.

## 3. Result

**Figure 1:**
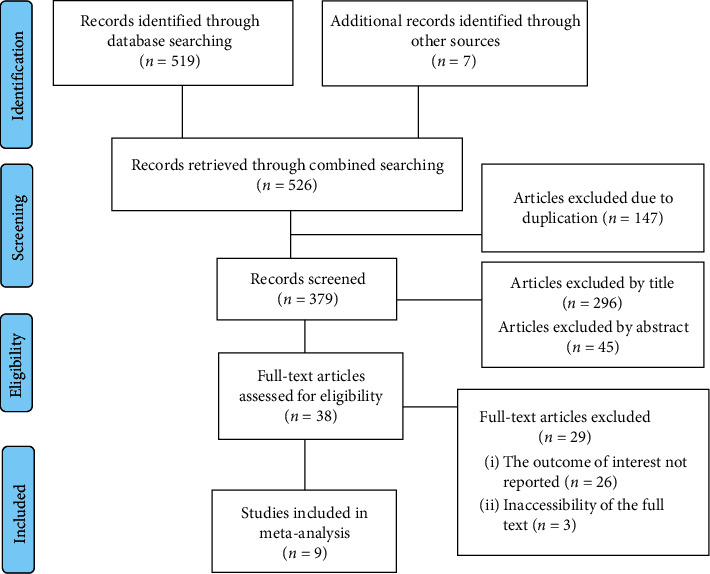
Flow chart of selection for systematic review and meta-analysis on the level of knowledge of nurses about palliative care and associated factors in Ethiopia.

In this review, a total of 526 articles were retrieved using different searching databases. Of all retrieved articles, 147 articles were excluded from the analysis due to duplication. From the remaining 379 studies, 341 were further excluded after critically reviewing the titles and abstracts. Furthermore, 29 articles were excluded which did not fulfill the inclusion criteria. Finally, nine articles were included in the final analysis (Figure 1).

### 3.1. Characteristics of the Studies and Study Participants

In this systematic review and meta-analysis, a total of nine articles were included with a total study population of 2709 (1137 males and 1572 females). The studies were three from the Amhara region [20–22], two from the Tigri region [23, 24], two from Addis Ababa city administration [25, 26], one from the Oromia region [27], and one from the Harar region [28]. All studies were cross-sectional in design and the sample size of the included studies ranged from 197 to 392 (Table 2).

### 3.2. Nurses' Knowledge about Palliative Care

**Figure 2:**
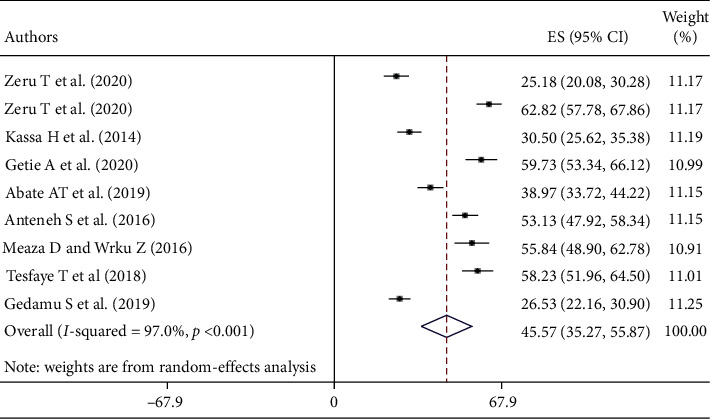
Forest plot of the pooled prevalence of nurses' knowledge about palliative care and associated factors in Ethiopia.

This systematic review and meta-analysis revealed that the pooled prevalence of nurses' knowledge about palliative care in Ethiopia was 45.57% (95% CI: 35.27–55.87) (Figure 2).

### 3.3. Heterogeneity and Subgroup Analysis

Heterogeneity was detected within the studies included in this review (*I*^2^ = 97.0%, *p* < 0.001). Therefore, subgroup analysis by a region where the study was conducted, the study period, and the sample size was carried out to detect the source of heterogeneity. The pooled prevalence of nurses' knowledge about palliative care was higher among nurses working in North Ethiopia, 47.93% (95% CI: 33.87–62.00). Similarly, the level of nurses' knowledge about palliative care was higher among studies conducted after 2017, 48.94% (95% CI: 34.01–63.87), and studies conducted before 2017, 41.37% (95% CI: 26.82–55.91). Higher prevalence of nurses' knowledge was reported among those studies with a sample size of <300, 49.67% (95% CI: 31.65–67.68) (Table 3).

### 3.4. Publication Bias

**Figure 3:**
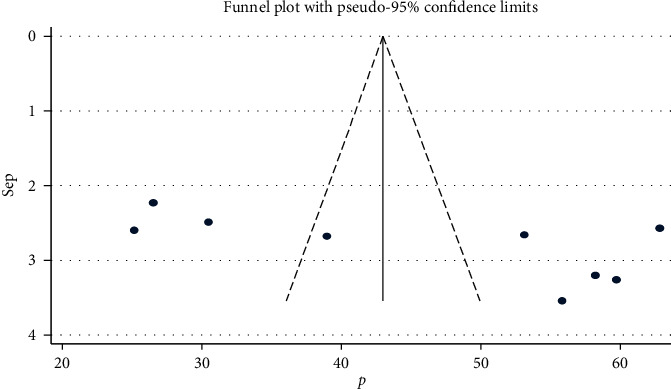
Funnel plot with 95% confidence limits of the pooled prevalence of nurses' knowledge about palliative care and associated factors in Ethiopia.

The results of this systematic review and meta-analysis showed that there is a symmetrical distribution of studies in the funnel plot and Egger's test was statistically insignificant (*p*=0.06), suggesting the absence of publication bias (Figure 3).

### 3.5. Sensitivity Analysis

The results of this study showed that none of the point estimates outside of the overall 95% confidence interval confirm that there is no influential study (Table 4).

### 3.6. Distribution of Nurses' Level of Education, Work Experience, and Training on Palliative Care

This systematic review and meta-analysis reported the pooled prevalence of level of education, work experience, the experience of giving care for chronically ill patients, and training on palliative care of nurses working in Ethiopia. Accordingly, more than half of the study participants were B.S. degree holder nurses, 68.96% (95% CI: 67.27–70.66). Regarding work experience, the majority of nurses had work experience of less than five years, 58.22% (95% CI: 56.52–59.92), and 50.87% (95% CI: 48.70–53. 05) of the nurses had the daily experience of giving care for chronically ill patients. Of all study participants included in this review, only 36.02% (95%CI: 34.06–37.98) were trained on palliative care (Table 5).

### 3.7. Factors Associated with the Knowledge of Nurses about Palliative Care

**Figure 4:**
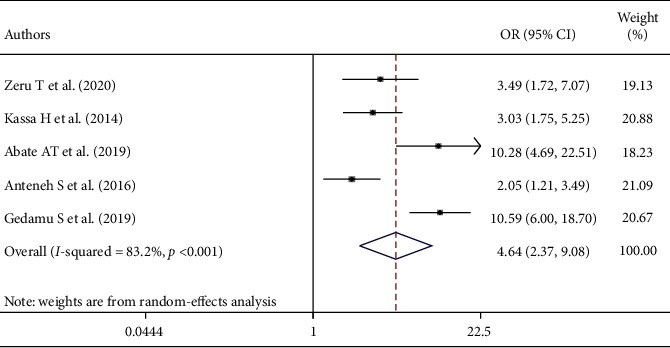
The overall pooled odds ratio of the association between training on palliative care and nurses' level of knowledge about palliative care in Ethiopia.

This systematic review and meta-analysis showed that there is a significant association between training on palliative care and nurses' level of knowledge about palliative care. The odds of nurses' knowledge were 4.64 times more likely among nurses who were trained on palliative care than their counterparts (AOR = 4.64; 95% CI: 2.37–9.08) (Figure 4).

**Figure 5:**
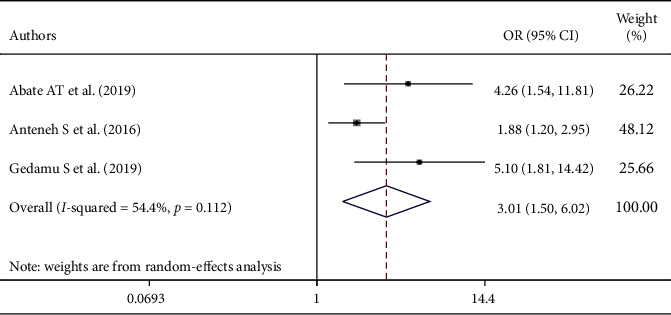
The overall pooled odds ratio of the association between educational status and nurses' level of knowledge about palliative care in Ethiopia.

This study also showed that there is an association between the level of education and nurses' level of knowledge about palliative care. The odds of nurses' knowledge were 3.01 times more likely among B.S. degree holder nurses than diploma nurses (AOR = 3.01; 95% CI: 1.50–6.02) (Figure 5).

## 4. Discussion

Providing palliative care should be a key component of the healthcare system that all healthcare organizations should strive to improve. Despite the understanding of the benefits of palliative care, many people living with chronic life-threatening illnesses do not receive palliative care. The primary challenges to apply palliative care are an overestimation of patient progress by health professionals and a low level of knowledge about palliative care. In this systematic review and meta-analysis, the pooled prevalence of the level of nurses' knowledge about palliative care in Ethiopia was 45.57% (95% CI: 35.27–55.87). This finding is almost similar to those in studies conducted in Pakistan (43.5%) [11], Greece (44.5%) [9], South Korea (48.3%) [29], Mongolia (40%) [10], Iran (39.3%) [3], Manipur (38%) [30], Saudi Arabia (36.5%) [31], India (38%) [2], Sudan (50.9%) [32], Spain (54%) [33], and South Iran (37.95%) [34]. Conversely, it is higher than those in studies done in Udupi district, India (20.5%) [35], Palestine (20.8%) [36], and Guwahati city, India (21%) [37]. The possible reason for this variation might be the integration of palliative care training with routine in the current study. The reason might be also the increase of chronic and life-threatening illness causing nurses to be exposed to giving palliative care or the variation in study participants in which the study participants of the previous studies were nursing students and all health professionals, whereas the current study includes nurses as a study subject. However, this finding is lower than that in a study done in India (67.6%) [38]. The reason might also be the variation in study setting, level of education, education about palliative care, and training on palliative care.

In the subgroup analysis, there was a variation in the level of nurses' knowledge about palliative care in the region where the studies were conducted, study period, and sample size. The higher prevalence of knowledge was reported among studies done in Northern Ethiopia, which might be due to the fact that most of the studies conducted in Northern Ethiopia were in referral hospitals. Similarly, the level of knowledge of nurses about palliative care was higher among studies conducted after 2017. This might be because the nurses included in the recent studies were trained about palliative care and most of them are B.S. degree holders.

Nurses with B.S. degree in their educational status were 3.01 times more likely knowledgeable about palliative care than diploma holders. This result is supported by studies conducted in the Northeastern region of the United States and Iran [3, 7]. This is because as the educational status of the nurses increases, their knowledge regarding palliative care is also increased due to an increment of professional knowledge, skills, and exposure to patients who need palliative care. The odds of nurses' knowledge were 4.64 times more likely among nurses who were trained on palliative care than those who were not trained on palliative care. This finding was in agreement with studies conducted in Iran, Greece, and Spain [3, 9, 31]. The possible reason might be the fact that getting updated training on palliative care raises the knowledge of nurses on palliative care.

## 5. Conclusion

This study revealed that more than half of the nurses had poor knowledge about palliative care. In this regard, the educational status of nurses and palliative care training were significantly associated factors with the nurses' level of knowledge about palliative care. Therefore, there should be incorporation of palliative care in the nursing curriculum. Furthermore, palliative care training and continuous education should be given regularly for nurses to improve their knowledge about palliative care.

### 5.1. Strength and Limitations of the Study

This review covers a wide area and accessed different articles making the review more accurate, and a subgroup and sensitivity analysis was carried out, which overcomes heterogeneity and checks the presence of influential studies. However, this study includes only studies with a cross-sectional design, which may be limited to generate a cause-effect link. It also sticks to the meta-analysis of observational studies in epidemiology.

## Figures and Tables

**Table 1:** Searches from different databases about the level of knowledge and associated factors regarding palliative care among nurses in Ethiopia.

Databases	Searching terms	Number of studies
MEDLINE/PubMed	“Knowledge” OR “awareness” AND “palliative care” OR “end of life care” OR “caring terminally ill” AND “factors” OR “associated factors” OR “determinant factors” AND “nurses” OR “hospital-based nurses” AND “Ethiopia”	88
Google Scholar	“Knowledge” AND “palliative care” AND “associated factors” OR “determinant factors” AND “nurses” AND “Ethiopia”	431
Other databases		7
Total retrieved articles		526
Included studies		9

**Table 2:** Study characteristics included in the review and meta-analysis on nurse's knowledge about palliative care and associated factors in Ethiopia.

Region	Sample size	Level of education		Experience in caring chronically ill patients					Palliative care training		Knowledge (%)	NOS
		Diploma	B.S.	Daily	Once/week	Once/month	Few/year	Never	Yes	No		
Tigri	278	120	158	115	62	22	25	54	—	—	25.18	8
Tigri	355	169	186	165	79	17	40	54	267	88	62.82	8
Addis Ababa	341	170	171	186	70	27	33	25	74	267	30.50	9
Amhara	226	93	133	80	27	39	36	44	59	167	59.73	8
Amhara	331	45	286	153	78	48	4	48	62	269	38.97	7
Amhara	352	148	204	—	—	—	—	—	—	—	53.13	7
Harar	197	—	—	—	—	—	—	—	58	139	55.84	8
Oromia	237	106	131	—	—	—	—	—	—	—	58.23	8
Addis Ababa	392	57	335	271	56	18	37	10	112	280	26.53	8

NOS : Newcastle-Ottawa Scale; B.S. : Bachelor of Science.

**Table 3:** Subgroup analysis on the level of nurse's knowledge about palliative care and associated factors in Ethiopia (*n* = 9).

Variables	Subgroup	Studies (*n*)	Population	Prevalence (95% CI)	*I* ^2^ (%)	*p* value
Region	Northern Ethiopia	5	1542	47.93 (33.87–62.00)	97.1	<0.001
	Southern Ethiopia	4	1167	42.66 (26.78–58.45)	97.0	<0.001
Study period	≥2017	5	1427	48.94 (34.01–63.87)	97.2	<0.001
	<2017	4	1282	41.37 (26.82–55.91)	96.8	<0.001
Sample size	<300	4	938	49.67 (31.65–67.68)	97.1	<0.001
	≥300	5	1771	42.36 (28.71–56.01)	97.4	<0.001

Northern Ethiopia: Tigri region and Amhara region. Southern Ethiopia: Addis Ababa city administration, Oromia region, and Harar region.

**Table 4:** Sensitivity analysis of nurse's knowledge about palliative care and associated factors in Ethiopia.

Study omitted	Publication year	Estimated prevalence	(95% CI)
Zeru *T* et al.	2020	48.13	(37.62–58.64)
Zeru *T* et al.	2020	43.38	(33.22–53.55)
Kassa H et al.	2014	47.47	(36.31–58.64)
Getie a et al.	2020	43.82	(33.00–54.65)
Abate AT et al.	2019	46.41	(34.75–58.07)
Anteneh S et al.	2016	44.63	(33.27–55.99)
Meaza *D* and wrku Z	2015	44.32	(33.25–55.38)
Tesfaye *T* et al	2018	44.01	(33.07–54.94)
Gedamu S et al.	2017	47.98	(37.52–58.43)
Overall		45.57	(35.27–55.87)

CI : confidence interval.

**Table 5:** Distribution of nurse's level of education, work experience, and training on palliative care among nurses in Ethiopia.

Valuables	Classifications	Studies	Population	Prevalence (95% CI)	*I* ^2^ (%)	*p* value
Level of education	Diploma	8	908	31.04 (29.34–32.75)	97.8	<0.001
	B.S.	8	1604	68.96 (67.27–70.66)	97.8	<0.001
Work experience	<5 years	8	1398	58.22 (56.52–59.92)	95.1	<0.001
	5–10 years	8	648	25.57 (23.86–27.27)	40.3	0.110
	≥10 years	8	466	14.46 (13.12–15.79)	95.7	<0.001
Experience on giving care for chronically ill patient	Daily	6	970	50.87 (48.70–53.05)	95.1	<0.001
	Once/week	6	372	18.49 (16.76–20.21)	80.5	<0.001
	Once/month	6	171	07.18 (6.04–08.32)	87.9	<0.001
	Few times/year	6	175	04.72 (03.80–05.65)	94.8	<0.001
	Never	6	235	07.50 (06.36–08.65)	96.6	<0.001
Training on palliative care	Trained	6	632	36.02 (34.06–37.98)	98.9	<0.001
	Not trained	6	121	66.35 (64.40–68.29)	98.8	<0.001

## Data Availability

All the related data have been presented within the manuscript. The dataset supporting the conclusions of this article is available from the authors upon request.

## Abbreviations

AJO: African Journals OnLine
AOR: Adjusted odds ratio
B.S.: Bachelor of Science
CI: Confidence Interval
NOS: Newcastle-Ottawa Scale
PRISMA: Preferred Reporting Items for Systematic Review and Meta-analysis.

## Supplementary Materials

Supplementary Materials Table S1: Preferred Reporting Items for Systematic Review and Meta-analysis guideline for reporting of the findings.
